# Visualising structural and functional characteristics distinguishing between newly diagnosed high‐tension and low‐tension glaucoma patients

**DOI:** 10.1111/opo.13129

**Published:** 2023-03-25

**Authors:** Daniel Rafla, Sieu K. Khuu, Sahana Kashyap, Michael Kalloniatis, Jack Phu

**Affiliations:** ^1^ Centre for Eye Health The University of New South Wales Sydney New South Wales Australia; ^2^ School of Optometry and Vision Science The University of New South Wales Sydney New South Wales Australia; ^3^ School of Medicine (Optometry) Deakin University Victoria Geelong Australia; ^4^ Faculty of Medicine and Health University of Sydney New South Wales Camperdown Australia; ^5^ Concord Clinical School Concord Repatriation General Hospital New South Wales Concord Australia

**Keywords:** normal‐tension glaucoma, open‐angle glaucoma, optical coherence tomography, perimetry, primary open‐angle glaucoma, visual fields

## Abstract

**Purpose:**

To determine whether there are quantifiable structural or functional differences that can distinguish between high‐tension glaucoma (HTG; intraocular pressure [IOP] > 21 mm Hg) and low‐tension glaucoma (LTG; IOP ≤ 21 mm Hg) at diagnosis.

**Method:**

This was a retrospective, cross‐sectional study. Clinical results of one eye from 90 newly diagnosed HTG and 319 newly diagnosed LTG patients (117 with very‐low‐tension glaucoma [vLTG; ≤15 mm Hg] and 202 with middling LTG [mLTG; >15 mm Hg, ≤21 mm Hg]) were extracted, which included relevant demographic covariates of glaucoma, quantitative optical coherence tomography (including the optic nerve head, retinal nerve fibre layer and ganglion cell‐inner plexiform layer) measurements and standard automated perimetry global metrics. We used binary logistic regression analysis to identify statistically significant clinical parameters distinguishing between phenotypic groups for inclusion in principal component (PC) (factor) analysis (PCA). The separability between each centroid for each cohort was calculated using the Euclidean distance (*d*(*x*,*y*)).

**Results:**

The binary logistic regression comparing HTG and all LTG identified eight statistically significant clinical parameters. Subsequent PCA results included three PCs with an eigenvalue >1. PCs 1 and 2 accounted for 21.2% and 20.2% of the model, respectively, with a d(*x*,*y*) = 0.468, indicating low separability between HTG and LTG. The analysis comparing vLTG, mLTG and HTG identified 15 significant clinical parameters, which were subsequently grouped into five PCs. PCs 1 and 2 accounted for 24.1% and 17.8%, respectively. The largest separation was observed between vLTG and HTG (d(*x*,*y*) = 0.581), followed by vLTG and mLTG (d(*x*,*y*) = 0.435) and lastly mLTG and HTG (d(*x*,*y*) = 0.210).

**Conclusion:**

Conventional quantitative structural or functional parameters could not distinguish between pressure‐defined glaucoma phenotypes at the point of diagnosis and are therefore not contributory to separating cohorts. The overlap in findings highlights the heterogeneity of the primary open‐angle glaucoma clinical presentations among pressure‐defined groups at the cohort level.

**Key points**

- There are some differences in quantitative structural and functional parameters between newly diagnosed high‐ and low‐tension glaucoma.
- After considering all patients along the same spectrum of disease severity, there is no appreciable separation between pressure‐defined groups.
- There is substantial heterogeneity among structural and functional results of patients newly diagnosed with glaucoma at different intraocular pressure levels.

## INTRODUCTION

The glaucoma family of diseases represent a diverse spectrum of clinical presentations, united by a common constellation of clinical features and the risk of irreversible blindness.^1^, ^2^ The key clinical features of glaucoma include observable structural (at the optic disc and adjacent retinal nerve fibre layer [RNFL]) and functional (such as visual field [VF]) changes. Whilst chronic open‐angle glaucoma encompasses all presentations with varying combinations of structural and functional defects, several clinical phenotypes have been proposed that may have differences in natural history, management and prognosis.^3^

One method for distinguishing primary open‐angle glaucoma phenotypes is by the intraocular pressure (IOP) at baseline prior to treatment. Typically, this is divided into so‐called high‐tension glaucoma (HTG) and low‐tension glaucoma (LTG) (also referred to as normal‐tension glaucoma (NTG) in other investigations). Several seminal studies^3^, ^4^, ^5^, ^6^ have cited 21 mm Hg as the upper limit of, above which a patient would then be considered to have high‐tension glaucoma. The intraocular pressure phenotypes are notably significant as they have been reported to have differences in natural history, risk of progression and disease management, such as that described in the Early Manifest Glaucoma Trial (EMGT).^3^, ^6^, ^7^ Additionally, different levels of intraocular pressure within low‐tension glaucoma (either above or below 15 mm Hg)^8^, ^9^, ^10^ have been suggested to be subtly important in prognostication. For example, Kim et al.^11^ found that low‐tension glaucoma patients with intraocular pressure at or below 15 mm Hg demonstrated localised retinal nerve fibre layer defects closer to the macula compared with high‐teen levels, thus suggesting a different clinical presentation. Similarly, Ha et al.^12^ found that patients with lower intraocular pressure tended to have localised retinal nerve fibre layer defects closer to the macula, compared with patients with relatively higher intraocular pressure. Xu et al.^13^ described some differences in structural deficits between glaucoma groups in the peripapillary region, but interestingly no significant difference in the parafoveal region. Several studies have suggested that high‐tension glaucoma and low‐tension glaucoma phenotypes present with different patterns of optic nerve and VF changes, with high‐tension glaucoma presenting with more diffuse structural and peripheral functional loss, and low‐tension glaucoma presenting with more focal structural and central functional loss.^11^, ^14^ However, generalisation of structural and functional clinical parameters occurring in high‐ and low‐tension glaucoma remains equivocal. Previous studies such as that by Shin et al.^15^ and Konstantakopoulou et al.^16^ showed no significant differences in patterns of structural loss. In combination, there remains motivation to examine high‐ and low‐tension glaucoma phenotypes in terms of their clinical characteristics.

Historically, the use of IOP cut‐off criteria has been largely based on prior population studies,^17^ or median split of seminal studies such as the EMGT.^3^, ^6^ Thus, historically, a distinction between high‐ and low‐tension glaucoma became accepted in the clinical dogma.^1^ This raises the question of the applicability of this cut‐off criterion with respect to structural and functional differences between pressure‐defined phenotypes. This is also in the context of known variability in intraocular pressure measurements and variability (within and between visits) potentially differing between glaucoma and non‐glaucoma groups.^18^ Understanding phenotypic differences is important in optimising the management plan for the patient and providing an accurate prognosis.

The purpose of the present study was to determine whether quantitative differences in structural (on optical coherence tomography [OCT]) or functional (on standard automated perimetry [SAP]) features exist in patients with high‐ or low‐tension glaucoma at the time of diagnosis. The purpose was not to use structural or functional parameters to distinguish between pressure‐defined groups but rather to identify other potential structural or functional differences between the groups that may eventuate into changes in the management plan, and whether in addition to intraocular pressure‐related differences, there exists a spectrum of structural and/or functional change. We sampled patients attending a university‐based glaucoma service and identified those with a new diagnosis of glaucoma, separating them into high‐ and low‐tension glaucoma using 21 mm Hg as the upper limit of low‐tension glaucoma for all patients. We further subdivided all low‐tension glaucoma patients into two further sub‐categories, defined as very low‐tension glaucoma (vLTG) (≤15 mm Hg) and middling low‐tension glaucoma (mLTG) (>15, ≤21 mm Hg) as per a previously used criterion by Kim et al.^11^ We used a binary logistic regression analysis to identify parameters which were significant in separating phenotypes. We then applied a principal component (factor) analysis to identify and group correlated parameters into principal components. These principal components could then be used to create a quantitative score and to visualise the separation between high‐tension and low‐tension cohorts. In essence, principal components analysis places all data points (subjects) along a continuum (defined by the structural and functional variables) and allows visualisation of where each lies within the cohort. In doing so, it may be possible to identify the positions of subjects within each intraocular pressure‐defined cohort, and thus whether structural and functional differences at the cohort level exist.

## METHODS

### Study design and glaucoma diagnosis

This was a retrospective cross‐sectional study of patient files seen at the Centre for Eye Health, University of New South Wales. The study adhered to the tenets of the Declaration of Helsinki. Ethics approval was provided by the Human Research Ethics Committee of the University of New South Wales (approval number: HC200528). Patients provided written informed consent prior to participation in the study.

The files of patients who were referred to the Centre for Eye Health by an external optometrist for glaucoma assessment were extracted. The Centre for Eye Health is based within the University of New South Wales, Sydney, and is a referral‐only clinic that provides diagnostic imaging and treatment services for patients suspected of having diseases of the visual pathway. The glaucoma examination protocols of the Centre for Eye Health have been described previously.^19^, ^20^, ^21^, ^22^ In short, the examination includes the following: comprehensive history, visual acuities, anterior segment examination, applanation tonometry, pachymetry (Pachmate DGH55; dghtechnology.com/), gonioscopy, dilated stereoscopic examination of the optic nerve head and the macula, standard automated perimetry, colour fundus photography of the optic disc (stereoscopic) and posterior pole, optical coherence tomography imaging of the optic nerve head, retinal nerve fibre layer (RNFL) and macula (ganglion cell‐inner plexiform layer, GCIPL from the ganglion cell analysis) using the Cirrus OCT (Carl Zeiss Meditec, zeiss.com).

The records of all patients who attended the clinic from 2015 to 2019 for an initial glaucoma examination were manually extracted by reviewing appointments within the calendar year (1 January–31 December, inclusive). The purpose of using a single initial consultation was to assess the parameters as they appeared at the initial diagnosis of glaucoma, especially the pre‐treatment intraocular pressure level. As per the clinical protocols of the Centre for Eye Health, the diagnosis of glaucoma was made by the examining optometrist and would be reviewed by a senior optometrist or ophthalmologist to arrive at the final clinical diagnosis made at the time of the consultation. A third clinician (one of the study investigators who was not involved in the initial glaucoma diagnosis) reviewed the patient files to ensure consistency in the final diagnosis used for study purposes.^23^

Glaucoma diagnosis was confirmed by the presence of characteristic signs of optic nerve head damage, including but not limited to increased cup‐to‐disc ratio, cup‐to‐disc asymmetry, neuroretinal rim thinning, notching, cup widening and cup widening not attributed to other retinal or neurologic conditions and adjacent retinal nerve fibre layer thinning with or without the presence of concordant visual field defects found using the 24‐2 SITA‐Standard or 24‐2 SITA‐Faster algorithms (Humphrey Field Analyzer, zeiss.com). Patients with pre‐perimetric glaucoma with evidence of sufficient damage to warrant treatment were included in the study, as per the guidelines of the American Academy of Ophthalmology.^1^ Intraocular pressures were measured using applanation tonometry, and gonioscopy was performed to confirm that the iridocorneal drainage angle was open. Multiple intraocular pressure measurements were taken at the same clinic visit (with the highest reading taken as the baseline on the patient's medical record), but diurnal measurements within or across visits were not performed. As we used cross‐sectional data at the point of glaucoma diagnosis, we did not require the inclusion of confirmatory visual field defects (as the absence of visual field defects could still indicate pre‐perimetric glaucoma) nor overt disease progression. We did not include 10‐2 visual field data as not all patients routinely undertake 10‐2 testing in the clinic (thus, patients were ‘pre‐perimetric’ when using the 24‐2 test grid). The present definition, therefore, reflects a clinical paradigm based on current clinical guidelines,^1^ rather than more stringent, research‐based criteria.

For our initial analysis, patients were first split into binarised groups using commonly cited definitions from previous seminal studies.^3^, ^6^, ^7^, ^24^ In our study, we defined high‐tension glaucoma as having intraocular pressures > 21 mm Hg and low‐tension glaucoma as having intraocular pressures ≤ 21 mm Hg glaucoma groups as per the conventional clinical definition, which itself is based on historical precedent and is an accepted clinical principle.^1^ We did not use corrected intraocular pressure (such as using corneal thickness) due to the heterogeneity of adjustment methods and agreement of resultant intraocular pressure,^25^ and as methods for measuring other corneal biomechanical parameters were not available in the clinic. We also did not measure the translaminar pressure gradient as it is not routinely performed in the clinic. To further examine the clinical differences across the intraocular pressure spectrum, a secondary analysis was conducted using a trinary approach based on previous studies which have examined clinical profiles of high‐teen and low‐teen intraocular pressure.^9^, ^11^ We defined the three groups for this secondary analysis as high‐tension glaucoma (>21 mm Hg), middling low‐tension glaucoma (>15, ≤21 mm Hg) and very low‐tension glaucoma (≤15 mm Hg). Patients were assigned to their respective glaucoma subgroup based on the intraocular pressure measured at the time of diagnosis. When considering the potential contributions of corneal biomechanical factors on intraocular pressure readings, it would be reasonable to assume that any differences in structural or functional results would be maximal when comparing the HTG and very low‐tension glaucoma groups. However, for the purposes of the study, we continue to report on the middling low‐tension glaucoma group to aid in the visualisation of potential continuum of differences.

Inclusion criteria included a diagnosis of primary open‐angle glaucoma, ≥18 years of age and visual acuity 0.30 logMAR (6/12) or better. We noted that no patient with glaucoma had visual acuity worse than 0.30 logMAR (6/12) due to glaucoma, but causes of reduced vision were more commonly attributable to other concurrent ocular pathologies (as described in the exclusion criteria below). The focus of the study was on primary open‐angle glaucoma separated by classic definitions of high‐ and low‐tension glaucoma. Thus, patients with evidence of secondary glaucoma, such as pseudoexfoliation, pigment dispersion, traumatic, uveitic and others, were excluded from this study. Similarly, patients with narrow‐ or closed‐angle glaucoma were excluded as the focus was open‐angle glaucoma.

Exclusion criteria included the presence of concurrent ocular or neurological pathology (such as epiretinal membrane, pathologic myopia, cerebrovascular accident and others) and cataracts if the best corrected visual acuity was worse than 0.30 logMAR (6/12). Other specific exclusion criteria included patients not consenting to research, having incomplete medical records or with a diagnosis where the three clinicians were not in agreement. Pre‐defined quantitative cut‐offs for automatic test reliability metrics such as optical coherence tomography scan image quality and visual field reliability indices (i.e., fixation losses, false positives and false negatives) were not used as exclusion criteria for the purposes of this study if the obtained results were sufficient for diagnosis, in order to reduce selection bias^26^, ^27^, ^28^ and to capture a more practical view of clinical usage of test outputs. It is also notable that quantitative cut‐offs have been debated in the literature,^26^, ^27^, ^28^ with more recent studies emphasising the need for expert clinical interpretation. However, test results that were obviously clinically unusable were not included in the analysis. Examples include an abnormally elevated hill of vision, lens/ring scotomata and inattention defects on VF testing and large media opacities, truncation or uncompensated eye movements on optical coherence tomography.

### Data extraction

**FIGURE 1:**
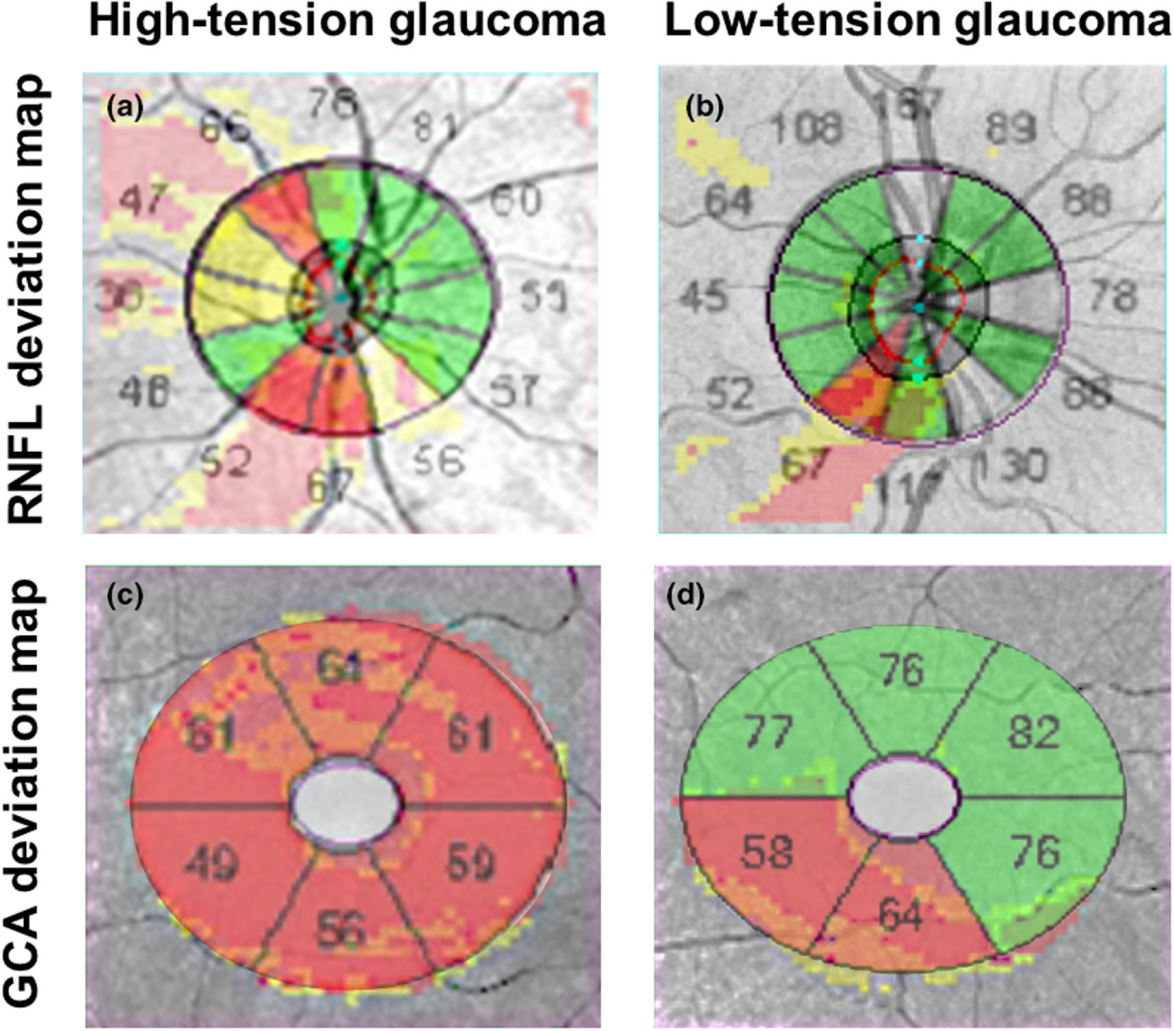
Examples of ‘classical’ structural findings^14^, ^34^ when distinguishing between binarised high‐tension glaucoma (HTG) and low‐tension glaucoma (LTG) phenotypes using a cut‐off intraocular pressure of 21 mm Hg. A patient with HTG showing more generalised glaucomatous damage (a and c) is compared with a patient with LTG with more focal inferotemporal glaucomatous damage (b and d). The retinal nerve fibre layer deviation maps are superimposed with clock hour results for HTG (a) and LTG (C) and the Ganglion Cell Analysis (GCA) deviation maps are superimposed with the sector maps for HTG (b) and LTG (d).

**FIGURE 2:**
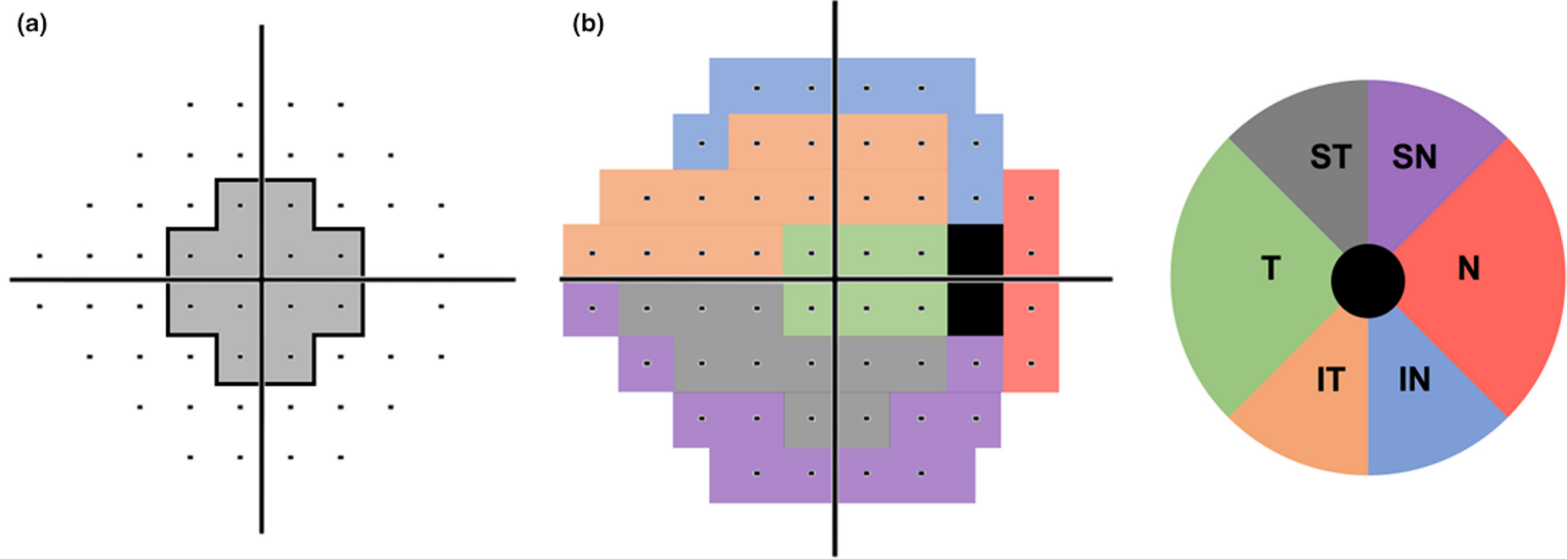
(a) 24‐2 grid with the 12 locations found within central 10° highlighted in grey zone. (b) Garway‐Heath visual field sector division of Bruch's membrane opening Garway‐Heath sector division map. IN, inferonasal; IT, inferotemporal; N, nasal; SN, superonasal; ST, superotemporal; T, temporal.

For all patients with bilateral glaucoma, one eye was randomly selected for the study, and where there was glaucoma in one eye only, then the affected eye was used. The following clinical data were extracted from the patient's medical record: age, self‐reported gender, self‐reported ethnicity (which included categories of Caucasian, East Asian, South Asian, Hispanic, Black, Pacific Islander and Indigenous Australian as per the clinical records of the Centre for Eye Health), visual acuity, spherical equivalent refractive error, intraocular pressure and central corneal thickness. For optical coherence tomography scans, the following parameters were extracted: average retinal nerve fibre layer thickness; retinal nerve fibre layer thicknesses for the superior, nasal, inferior and temporal quadrants; retinal nerve fibre layer thicknesses for each of the 12 clockfaces; disc symmetry; rim area; disc area; average and vertical cup‐to‐disc ratios; cup volume; average and minimum ganglion cell‐inner plexiform layer thickness; and ganglion cell‐inner plexiform layer thickness at the superotemporal, superior, superonasal, inferonasal, inferior and inferotemporal sectors (see Figure 1). For visual field parameters, Visual Field Index, mean deviation (MD) and pattern standard deviation (PSD), fixation losses and false positive rates were extracted. We also reported the number of test locations within the central 10° of the 24‐2 (described as the C24‐2 by previous studies) which exhibited a defective on pattern deviation map at *p* < 0.05 or lower, as per the methods reported by previous studies.^29^, ^30^, ^31^ This was performed in the absence of 10‐2 data to identify patients with a presentation of glaucoma which is potentially central‐affecting compared with peripheral‐affecting (a reported feature distinguishing high‐ and low‐tension glaucoma). These test locations corresponded to Cartesian coordinates for right eye orientation in the supero‐temporal quadrant of (3, 3), (3, 9), (9, 3) and their respective mirror locations in the respective quadrants of the 24‐2 test grid (see Figure 2a). This analysis focussed on central deficits that have been suggested to be more prevalent in low‐tension glaucoma. Additionally, we separated the entirety of the 24‐2 test grid into five sectors using the established Garway‐Heath sector maps.^32^, ^33^ This allowed us to identify potentially sector‐wise differences between cohorts. Analyses of region‐wise (either central or by sectors) were performed using the pattern deviation map of 24‐2, where we calculated the percentage of defective locations in each VF sector. A defective location was defined as being *p* < 0.05 or worse (see Figure 2b).

Additionally, we also included the presence of self‐reported medical conditions and other risk factors that may be relevant to glaucoma risk, including migraines, diabetes, hypertension, sleep apnoea, prolonged steroid use, smoking and family history of glaucoma.^3^, ^24^, ^35^ These categorical variables were binarised (present or absent).

### Logistic regression

Backward stepwise binary logistic regression analysis (SPSS Statistics software version 24, ibm.com) was used to identify variables that could distinguish between high‐ and low‐tension glaucoma groups. The fit of the model was assessed using Naglekerke *R* ^2^, with the statistical significance of contributing variables set at *p* < 0.05.

Variables included in the preliminary analysis, coded as scaled data, included all extracted quantifiable clinical parameters from the Cirrus optical coherence tomography and visual field testing as listed above, in addition to other quantitative parameters including spherical equivalent refractive error, age and central corneal thickness. As noted above, intraocular pressure was used to define the groups and thus was not used in the regression analysis. Additionally, the following variables were coded as categorical data and included in the analysis, namely self‐reported gender, history of migraines, diabetes, hypertension, sleep apnoea, prolonged steroid use, smoking and family history of glaucoma.

### Factor analysis

Factor (principal components) analysis (SPSS Statistics software version 25, ibm.com) was used to refine further the significant parameters identified by the preceding binary logistic regression model for both diagnoses into grouped principal components. The principal components are combinations of variables that are correlated and then ranked by their overall proportional contribution in explaining a difference between groups. Other supervised methodologies such as multivariate analysis that would require significant user input were considered. In this present study, we elected to use a partially supervised principal components analysis as our primary analysis to mitigate any potential user bias, and we removed any potential outlier results which may bias the interpretation of results. This was in part due to the known clinical utility of some clinical parameters for the diagnosis of glaucoma, compared with others that are comparatively low yield. The principal components in this study represent clinical variables that may be correlated in their contributions for explaining differences between high‐ and low‐tension glaucoma.^23^, ^36^, ^37^, ^38^

The process of principal components analysis has been described in our previous work^23^, ^28^ and has also been used in several other ophthalmological studies as a method for visualising distinctions between two cohorts.^39^, ^40^ In the present investigation, we extracted the individual patient results only for those clinical parameters deemed statistically significant using binary logistic regression analysis. These were in turn converted into a *Z*‐score relative to the distribution of the totality of the cohort. *Z*‐scores are used to standardise values across a variety of different parameters, with different units, such as retinal nerve fibre layer thickness in micrometres and visual field mean deviation in decibels. We used varimax rotation, which was used to determine the final rotated component matrices. Varimax component coefficients <0.4, representing weaker correlations, were excluded (in the context that no universally accepted cut‐off value exists). Components with an eigenvalue >1 were used to determine the prinicpal components, which more significantly contribute to the varimax rotation model. A cut‐off eigenvalue of more than one when using an accompanying subjective assessment of the scree plot has previously been suggested^41^, ^42^ to mitigate the overreporting of significant factors, compared with a more conservative approach such as a parallel analysis. Additionally, having the maximum number of factors would be useful at the start to determine whether models share similar characteristics.

For each patient, we calculated a factor score, which is a multiple of the *Z*‐score with its corresponding variable, summed within the principal component. The factor scores of principal components 1 and 2 for each patient were then plotted on a biplot, which allows the visualisation of overlap between individual data from both high‐tension and low‐tension groups, and thus the separability between both sub‐diagnoses. As described in the Introduction, the axes of the biplots represent the continuum of structural and/or functional parameters when considering the totality of the cohort, and each data point represents one subject. As the subjects are already known to belong to a pressure‐defined group, the ‘separation’ between high tension and low tension is examined at the cohort level to determine additional potential differences in their structural and functional characteristics.

To quantify the separability between ‘average’ patients within each group using principal components 1 and 2, the Euclidean distance (denoted as d(*x*,*y*)) was calculated using the formula $d\left(x,y\right)=\sqrt{{\left(p_{1}-q_{1}\right)}^{2}+{\left(p_{2}-q_{2}\right)}^{2}}$ where *P* (*p*_1_, *p*_2_) and *Q* (*q*_1_, *q*_2_) represent the two centroid locations (as per the *x* and *y* values) for each cohort. The Kaiser–Meyer–Olkin (KMO) statistic was used to assess sampling adequacy and was set at a cut‐off value >0.4.

### Statistical analyses

Basic demographic and clinical information were assessed first using the D'Agostino Pearson test to determine whether the continuous data were distributed normally, and Hedges' *g* was used to calculate the effect size between cohorts. ANOVA *t*‐tests and one‐way ANOVA tests were performed for continuous data with *p* < 0.05 considered to be significant.

## RESULTS

### Demographic characteristics

From 2015 to 2019, we reviewed 4808 files of patients who had been initially referred for glaucoma assessment. Of this group, 708 subjects were newly diagnosed with glaucoma. Of these, 299 subjects were excluded due to incomplete records or other exclusion criteria. Thus, 319 newly diagnosed low‐tension glaucoma (≤21 mm Hg) and 90 newly diagnosed high‐tension glaucoma (>21 mm Hg) patients were eligible for inclusion in the study. Table 1 describes the demographic characteristics identified between both high‐ and low‐tension glaucoma patients. The low‐tension glaucoma cohort was slightly older than high‐tension patients, but this was not statistically significant (*p* = 0.99). In this cohort, Caucasian patients were most prevalent in high‐tension glaucoma, and those of East Asian descent were the most prevalent with low‐tension glaucoma (*p* = 0.019). There were more males than females in both high‐ and low‐tension glaucoma cohorts (*p* = 0.72). As expected, mean intraocular pressure was found to be significantly higher in high‐tension glaucoma, 25.0 ± 3.3 mm Hg compared with low‐tension glaucoma 16.7 ± 2.7 mm Hg (*p* < 0.001).

**TABLE 1:** Demographic characteristics of the glaucoma cohorts.

Characteristics	Low tension (*n* = 319)	High tension (*n* = 90)	*p*‐Value
Age (years, mean and SD)	62.0 ± 11.4	61.1 ± 11.9	0.99
Self‐reported gender, *n* (%)			
Male	190 (59.6)	56 (62.2)	0.72
Female	129 (40.4)	34 (37.8)	
Self‐reported ethnicity, *n* (%)			
Caucasian	107 (33.5)	46 (51.1)	0.019^a^
East Asian	113 (35.4)	25 (27.8)	
South Asian	33 (10.3)	4 (4.4)	
Hispanic	4 (1.3)	4 (4.4)	
Black	1 (<0.1)	3 (3.3)	
Pacific Islander	1 (<0.1)	0 (0)	
Aboriginal	4 (1.3)	1 (1.1)	
Medical history, *n* (%)			
Migraine	23 (7.2)	8 (8.9)	0.42
Hypertension	96 (30.1)	30 (33.3)	0.39
Diabetes	31 (9.7)	12 (13.3)	0.26
Sleep apnoea	25 (7.8)	2 (2.2)	0.02^a^
Corticosteroid use	12 (3.8)	3 (3.3)	0.83
Smoking	72 (22.6)	25 (27.8)	0.83
Family history of glaucoma (first or second degree)	25 (7.8)	2 (2.2)	0.94
Spherical equivalent refraction (D, median, IQR)	−0.25 (−2.25 to +1.00)	−0.38 (−2.31 to 2.75)	0.30
IOP (mm Hg, median, IQR)	17.0 (15.0 to 19.0)	24.0 (22.9 to 26.0)	<0.0001^a^
Ganglion cell‐inner plexiform layer thickness (μm, median, IQR)	72.5 (66.8 to 78.0)	71.3 (67.7 to 77.3)	0.06
Number of central VF test locations exhibiting a defect at the *p* < 0.05 or worse level (out of 12, median, IQR)	5 (4 to 6)	5 (4 to 7)	0.72
VF mean deviation (dB, median, IQR)	−2.20 (−4.07 to −0.90)	−2.47 (−5.97 to −0.66)	0.62

Abbreviations: IOP, intraocular pressure; IQR, interquartile range; SD, standard deviation; VF, visual field.

^a^ Statistically significant differences.

### Pairwise comparison of quantitative differences between high‐ and low‐tension glaucoma cohorts

**FIGURE 3:**
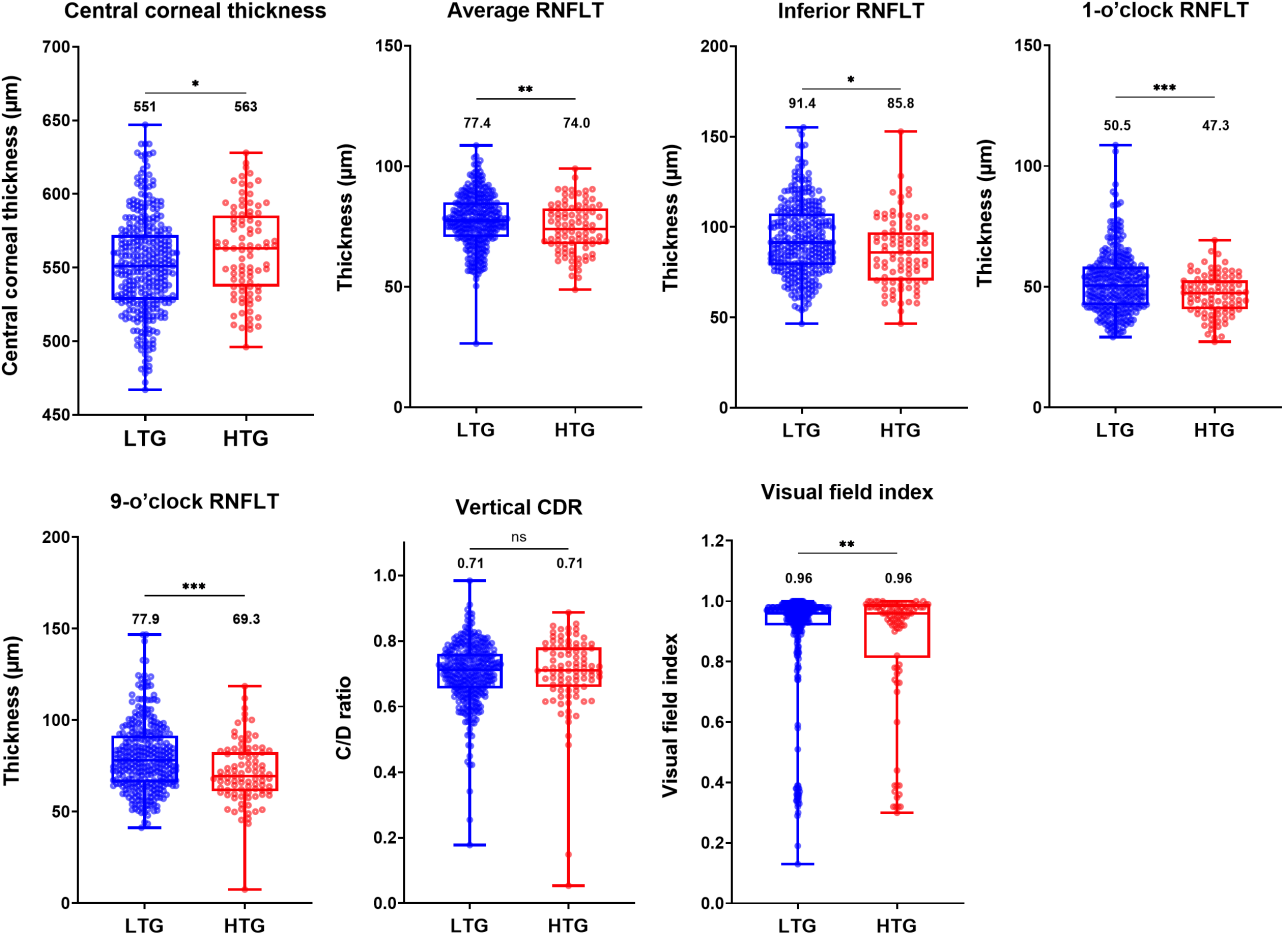
Box‐and‐whisker plots showing the median, interquartile and full‐range results of all quantitative clinical parameters for all low‐tension (LTG = blue) and high‐tension (HTG = red) glaucoma patients deemed statistically significant and included in factor analysis (*p < 0.05, **p < 0.01, ***p < 0.001). The values displayed represent the median result for each cohort. CDR, cup‐to‐disc ratio; RNFLT, retinal nerve fibre layer thickness.

For our initial analysis, we first chose to examine the differences between high‐ and low‐tension glaucoma subjects based on the more commonly used 21 mm Hg cut‐off criterion to compare against previously cited seminal studies such as the EMGT.^3^, ^6^, ^7^ We conducted a secondary analysis following this initial stage by further separating all low‐tension glaucoma patients into very low‐tension glaucoma (≤15 mm Hg) and middling low‐tension glaucoma (>15 mm Hg, and ≤21 mm Hg). We examined 36 clinical parameters and eight demographic parameters other than intraocular pressure comparing high‐ and low‐tension glaucoma cohorts (Figure S1). The binary logistic regression analysis identified eight parameters deemed statistically significant which would be included in the preliminary factor analysis (see Figure 3). These parameters included average, inferior, 1‐ and 9‐o'clock retinal nerve fibre layer thicknesses, central corneal thickness, vertical cup‐to‐disc ratio, Visual Field Index and sleep apnoea. When comparing the overall differences between high‐tension and low‐tension cohorts, there was a large degree of overlap between both cohorts in all clinical parameters, and the effect size was calculated as <0.5 for all significant clinical parameters. For instance, the average retinal nerve fibre layer thickness in high‐ and low‐tension glaucoma was 77.4 μm (interquartile range [IQR] 70.7–85.0 μm) and 74.0 μm (IQR 67.7–82.6 μm), respectively (*p* = 0.01). Notably, when examining the number of central locations exhibiting statistically significant defects at the *p* < 0.05 or worse level, the median results were very similar between high‐ (5, IQR 4–6) and low‐tension glaucoma cohorts (5, IQR 4–7) (*p* = 0.72).

### Principal component analysis: high‐ and low‐tension glaucoma

The binary logistic regression models extracted eight clinical parameters that were deemed statistically significant (Table 2).

**TABLE 2:** Quantitative clinical parameters of high‐tension and low‐tension glaucoma cohorts.

Characteristics, median (IQR)	Low tension (*n* = 319)	High tension (*n* = 90)	*p* Value
Central corneal thickness (μm)	551 (528–572)	563 (537–586)	0.02^a^
Average RNFL thickness (μm)	77.4 (70.7–85.0)	74.0 (67.7–82.6)	0.01^a^
Inferior RNFL thickness (μm)	91.4 (78.9–107.5)	85.8 (70.4–97.1)	0.05^b^
1 o'clock RNFL thickness (μm)	50.5 (42.4–58.3)	47.3 (40.7–52.7)	<0.001^a^
9 o'clock RNFL thickness (μm)	77.9 (66.0–91.6)	69.3 (61.1–82.5)	<0.001^a^
Vertical CDR	0.71 (0.65–0.76)	0.71 (0.66–0.78)	0.06^b^
Visual Field Index (%)	0.96 (0.92–0.98)	0.96 (0.81–0.99)	0.006^a^

*Note*: All *p* values were calculated using binary logistic regression model.

Abbreviations: CDR, cup‐to‐disc ratio; IQR, interquartile range; RNFL, retinal nerve fibre layer.

^a^ Statistically significant differences.

^b^ Included in factor analysis as a significant variable.

However, due to the small proportion of patients reporting a medical history of sleep apnoea, we conducted a principal components analysis with the other seven parameters (excluding sleep apnoea), which generated three principal components with an eigenvalue >1 for the final biplot (Table 3). Principal component 1 (consisting of average, inferior and 9‐o'clock retinal nerve fibre layer thicknesses and vertical cup‐to‐disc ratio) and principal component 2 (consisting of 1‐o'clock and central corneal thickness) accounted for approximately 38% of the variance of the model used to distinguish between high‐ and low‐tension glaucoma cohorts. The Kaiser‐Meyer‐Olkin result was 0.674, where a value of <0.8 suggests the result may contain inadequate variables, cases or both for factor analysis. However, a low Kaiser‐Meyer‐Olkin result may also occur when there is no significant difference between the two cohorts examined despite adequate power.^43^

**TABLE 3:** Rotated component matrices from factor analysis on high‐tension and all low‐tension glaucoma subjects.

Clinical parameters	PC‐1 (37.9%)	PC‐2 (15.3%)	PC‐3 (15.3%)
Average RNFL thickness (μm)	0.895	—	—
Inferior RNFL thickness (μm)	0.901	—	—
1‐o'clock RNFL thickness (μm)	—	0.476	−0.433
9‐o'clock RNFL thickness (μm)	0.850	—	—
Vertical CDR	−0.415	—	−0.411
Visual Field Index (%)	—	—	0.817
Central corneal thickness (μm)	—	0.880	—

*Note*: The percentage of variance accounted for by each principal component (PC) is shown in the header.

Abbreviations: CDR, cup‐to‐disc ratio; RNFL, retinal nerve fibre layer.

**FIGURE 4:**
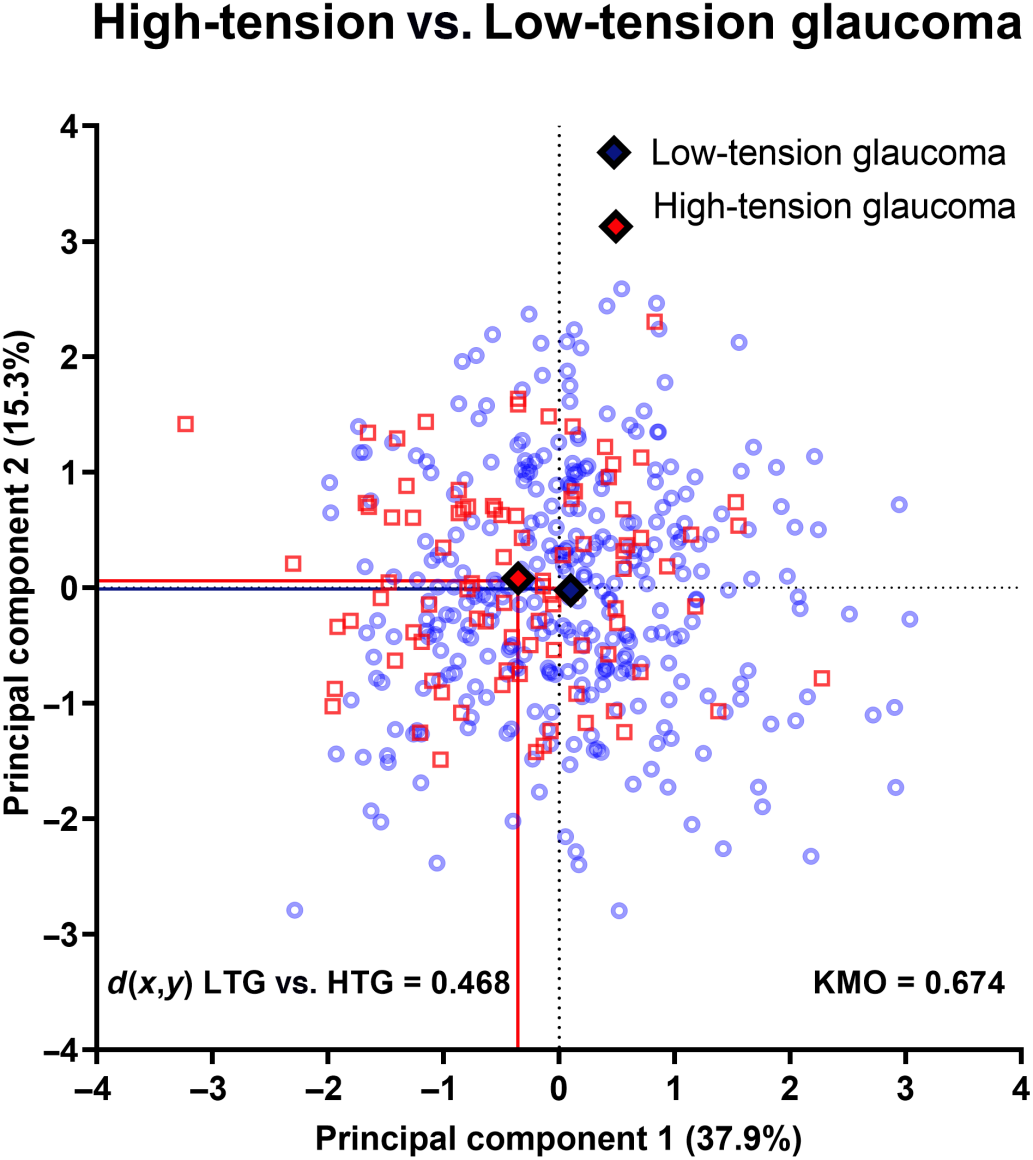
Biplot mapping individual patient results comparing principal components (PCs) 1 and 2, which have been demonstrated for clarity, where PCs 1 and 2 are shown on the *X*‐ and *Y*‐axes, respectively. Blue circles represent patients with low‐tension glaucoma and red squares indicate high‐tension glaucoma, with the solid diamonds representing the centroid for each PC for both cohorts. The separability between centroids (*d*(*x*,*y*)) is shown in the inset. The solid lines highlight the centroid results for each PC. KMO, Kaiser–Meyer–Olkin.

For simplicity, only principal components 1 and 2 (the largest contributors to the model using the seven parameters and excluding self‐reported sleep apnoea) were included in the biplot (Figure 4). For a full representation of principal components 1–3 using a three‐dimensional plot, see Figure S2. Figure 4 shows a substantial overlap between subjects in the high‐tension and low‐tension groups. The Euclidean distance between centroids was low at 0.468 (where Euclidean distance >1 indicates separability). Moreover, the average distance from the centroid for all low‐tension glaucoma subjects was 1.261 ± 0.687 and for high‐tension glaucoma subjects it was 0.760 ± 0.578 (*p* < 0.001). Thus, we observed that there was no clear separability between newly diagnosed high‐ and low‐tension glaucoma patients.

### Subdivision of the low‐tension glaucoma cohort

**FIGURE 5:**
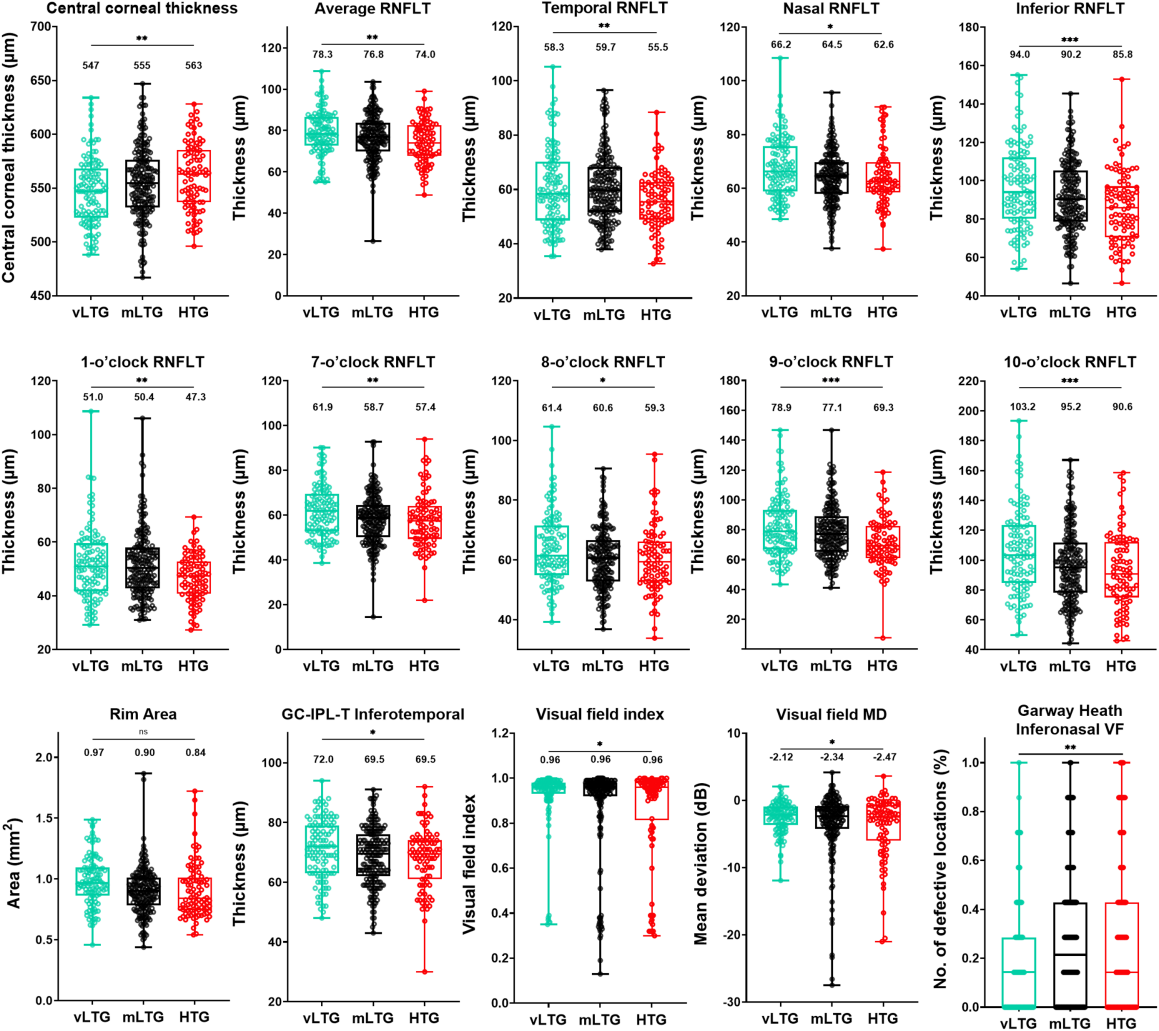
Box‐and‐whisker plot showing the median, interquartile and full range of individual results in all quantitative clinical parameters for very low‐tension glaucoma (vLTG = aqua), middling low‐tension glaucoma (mLTG = black) and high‐tension glaucoma (HTG = red) patients deemed statistically significant and included in the factor analysis (*p < 0.05, **p < 0.01, ***p < 0.001 for the multiple comparisons analysis in ANOVA). The value displayed represents the median result for each cohort. CDR, cup‐to‐disc ratio; GC‐IPL‐T, ganglion cell‐inner plexiform layer thickness; MD, mean deviation; RNFLT, retinal nerve fibre layer thickness; VF, visual field.

Considering the intraocular pressure cut‐off criteria which have been used to define two additional levels of low‐tension glaucoma, we conducted a subsequent analysis to examine whether there were quantitative clinical differences that could be observed between the lower and middling low‐tension glaucoma. For this analysis, we classified patients with IOP ≤15 mm Hg as very low‐tension glaucoma and between 15 and 21 mm Hg inclusive as middling low‐tension glaucoma. Mean intraocular pressures for the middling‐ and low‐tension glaucoma groups were 18.4 ± 1.7 mm Hg and 13.8 ± 1.7 mm Hg, respectively (*p* < 0.001). We conducted a one‐way ANOVA test to determine the quantitative clinical parameters which would be included in the principal components analysis, with statistical significance set at *p* < 0.05. The 15 parameters with statistically significant differences are shown in Figure 5 and Table 4 (for full results of all parameters, see Figure S3). While some of the parameters were similar to the binarised analysis (high‐ versus low‐tension glaucoma), several additional parameters were found to be significant in the trinary analysis, and these were included in the subsequent principal components analysis.

**TABLE 4:** Quantitative clinical parameters of high‐tension glaucoma (HTG), middling low‐tension glaucoma (mLTG) and very low‐tension glaucoma (vLTG).

Characteristics, median (IQR)	vLTG (≤15 mm Hg) (*n* = 117)	mLTG (15<; ≤21 mm Hg) (*n* = 202)	HTG (>21 mm Hg) (*n* = 90)	*p* Value
IOP (mm Hg)	14.0 (13.0 to 15.0)	18.0 (17.0 to 20.0)	24.0 (22.9 to 26.0)	<0.0001^a^
Central corneal thickness (μm)	547 (523 to 568)	555 (532 to 576)	563 (537 to 585)	0.01^a^
Average RNFL thickness (μm)	78.3 (72.7 to 86.5)	76.8 (69.9 to 83.8)	74.0 (67.7 to 82.6)	0.009^a^
Temporal RNFL thickness (μm)	58.3 (48.6 to 70.3)	59.7 (51.6 to 68.3)	55.5 (48.9 to 62.7)	0.009^a^
Nasal RNFL thickness (μm)	66.2 (58.8 to 75.6)	64.5 (57.9 to 69.8)	62.6 (58.4 to 69.7)	0.01^a^
Inferior RNFL thickness (μm)	94.0 (80.1 to 112.2)	90.2 (78.6 to 105.4)	85.8 (70.4 to 97.1)	<0.0001^a^
1‐o'clock RNFL thickness (μm)	51.0 (41.7 to 59.5)	50.4 (42.7 to 57.9)	47.3 (40.7 to 52.7)	0.002^a^
7‐o'clock RNFL thickness (μm)	61.9 (52.7 to 69.4)	58.7 (50.0 to 64.5)	57.4 (49.4 to 64.1)	0.006^a^
8‐o'clock RNFL thickness (μm)	61.4 (54.9 to 71.5)	60.6 (52.8 to 66.6)	59.3 (52.7 to 66.1)	0.02^a^
9‐o'clock RNFL thickness (μm)	78.9 (66.9 to 93.4)	77.1 (65.3 to 89.1)	69.3 (61.1 to 82.5)	<0.0001^a^
10‐o'clock RNFL thickness (μm)	103.2 (84.7 to 123.5)	95.2 (78.2 to 111.9)	90.6 (75.1 to 112.1)	<0.0001^a^
Rim area (mm^2^)	0.97 (0.86 to 1.10)	0.90 (0.78 to 1.01)	0.84 (0.74 to 1.01)	<0.001^a^
Inferotemporal GC‐IPL thickness (μm)	72.0 (63.0 to 79.0)	69.5 (62.0 to 76.0)	69.5 (61.0 to 74.0)	0.04^a^
Visual Field Index (%)	0.96 (0.93 to 0.98)	0.96 (0.92 to 0.98)	0.96 (0.81 to 0.99)	0.04^a^
VF mean deviation (dB)	−2.12 (−3.64 to −0.91)	−2.34 (−4.26 to −0.84)	−2.47 (−5.97 to −0.67)	0.04^a^
Garway‐Heath inferonasal VF sector (dB)	0.14 (0.14 to 0.22)	0.21 (0.24 to 0.32)	0.14 (0.22 to 0.36)	0.002^a^

*Note*: *p*‐Values calculated using one‐way ANOVA, where statistical analysis is set at *p* < 0.05.

Abbreviations: GC‐IPL, ganglion cell‐inner plexiform layer; IOP, intraocular pressure; IQR, interquartile range; RNFL, retinal nerve fibre layer; VF, visual field.

^a^ Statistically significant differences.

Subsequent principal components analysis grouped these parameters into five principal components with eigenvalue >1, where principal component 1 (consisting of average, inferior quadrant, 9‐ and 10‐o'clock retinal nerve fibre layer thicknesses, inferotemporal ganglion cell‐inner plexiform layer thickness and rim area) and principal component 2 (consisting of nasal, 7‐ and 8‐o'clock retinal nerve fibre layer thicknesses) accounted for 41.9% of the variance model (Table 5). The variance for this model was spread among more principal components. The Kaiser‐Meyer‐Olkin result for this analysis was 0.694.

**TABLE 5:** Rotated component matrices from factor analysis on very low‐tension, middling low‐tension and high‐tension glaucoma subjects.

Clinical parameters	PC‐1 (24.1%)	PC‐2 (17.8%)	PC‐3 (13.8%)	PC‐4 (11.8%)	PC‐5 (7.4%)
Average RNFL thickness (μm)	0.716	—	0.436	—	—
Inferior RNFL thickness (μm)	0.945	—	—	—	—
Inferotemporal GC‐IPL thickness (μm)	0.460	—	—	—	—
Rim area (mm^2^)	0.678	—	—	—	—
9‐o'clock RNFL thickness (μm)	0.777	—	—	—	—
10‐o'clock RNFL thickness (μm)	0.896	—	—	—	—
Nasal RNFL thickness (μm)	—	0.946	—	—	—
7‐o'clock RNFL thickness (μm)	—	0.852	—	—	—
8‐o'clock RNFL thickness (μm)	—	0.847	—	—	—
Temporal RNFL thickness (μm)	—	—	0.945	—	—
1‐o'clock RNFL thickness (μm)	—	—	0.931	—	—
VF mean deviation (dB)	—	—	—	0.931	—
Visual Field Index (%)	—	—	—	0.920	—
Central corneal thickness (μm)	—	—	—	—	0.774
Garway‐Heath Inferonasal VF sector (dB)					0.684

*Note*: The percentage of variance accounted for by each principal component (PC) is shown in the header.

Abbreviations: GC‐IPL, ganglion cell‐inner plexiform layer thickness; RNFLT, retinal nerve fibre layer thickness; VF, visual field.

**FIGURE 6:**
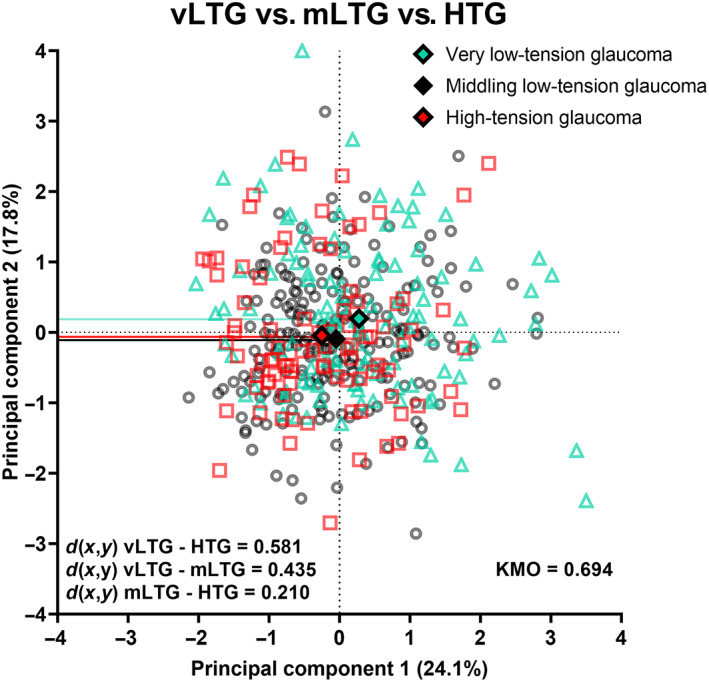
Biplot mapping individual patient results comparing principal components (PCs) 1 and 2, which have been demonstrated for clarity, where PCs 1 and 2 are shown on the *x*‐ and *y*‐axes, respectively. Light blue triangles represent very low‐tension (vLTG), black circles represent middling low‐tension (mLTG) and red squares represent high‐tension (HTG) glaucoma patients. Solid diamonds represent the centroid for both PCs for each cohort. The separability between centroids (*d*(*x*,*y*)) is shown in the inset. Solid lines highlight the centroid results for each PC. KMO, Kaiser–Meyer–Olkin.

As shown for the binarised analysis, principal components 1 and 2 were plotted on the biplot in Figure 6. We also presented principal components 1–3 in a three‐dimensional plot, to account for greater than 50% of the variance in Figure S4. There was substantial overlap observed between individual patient results for all three glaucoma cohorts, similar to the binarised categorisation of high‐ and low‐tension glaucoma. The Euclidean distance between high‐ and very low‐tension glaucoma was the highest (*d*(*x*,*y*) = 0.571), followed by that of very and middling low‐tension glaucoma (*d*(*x*,*y*) = 0.431) and lastly high‐ and middling low‐tension glaucoma being the shortest (*d*(*x*,*y*) = 0.204). Additionally, the average distance from the centroids for very low‐, middling low‐ and high‐tension glaucoma was 1.344 ± 0.770, 1.128 ± 0.623 and 1.247 ± 0.672, respectively (*p* = 0.03). Notably, the main difference between all three cohorts was observed along the *X*‐axis, representing principal component 1, which comprised only structural parameters. However, there was still no meaningful separation that could be observed between newly diagnosed very low‐, middling low‐ and high‐tension glaucoma.

**FIGURE 7:**
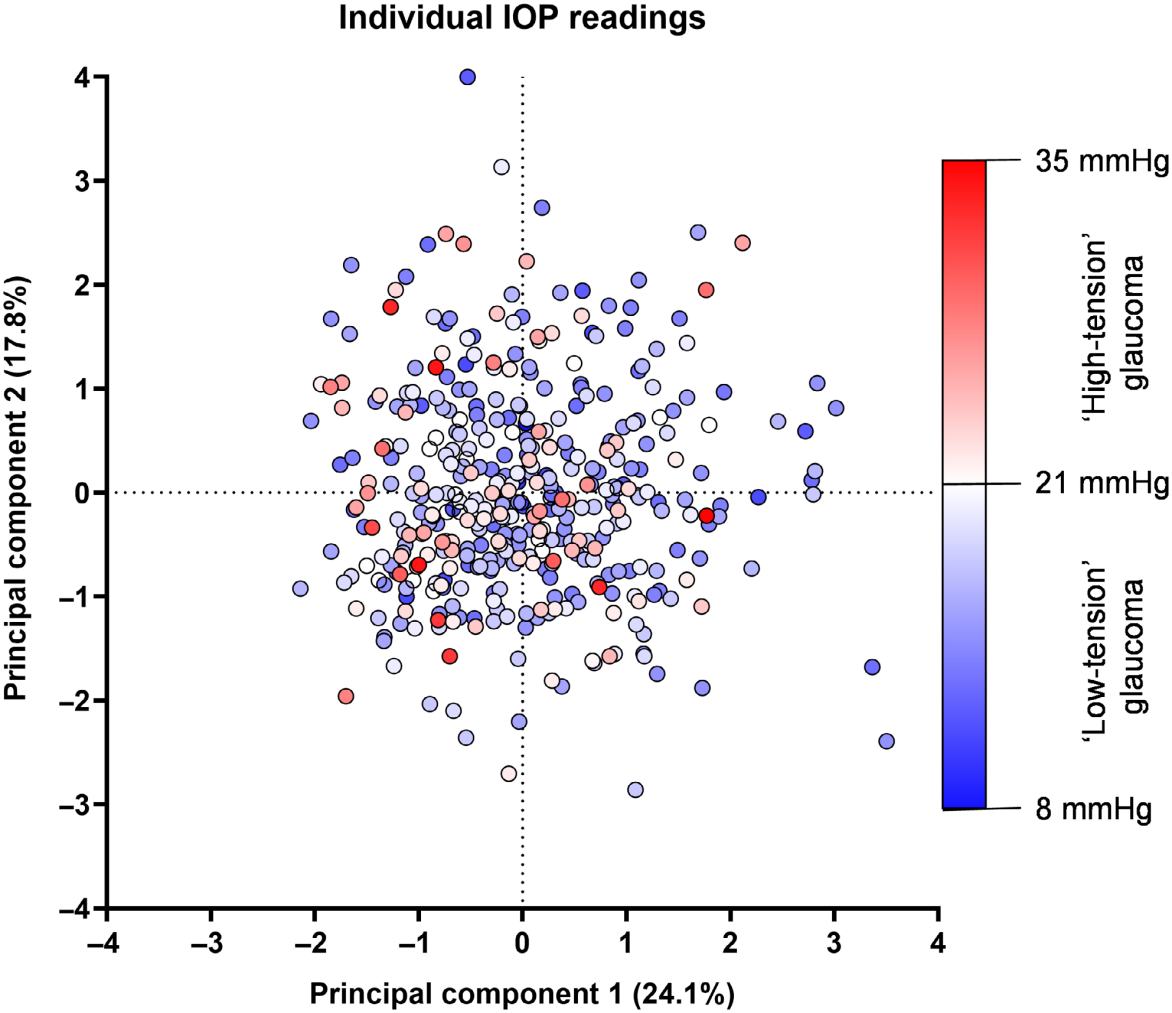
Biplot as per Figure 6, but with individual datum points coloured by their intraocular pressure (IOP) level rather than by discrete pressure‐defined groups. A colder/bluer colour indicates lower IOP (‘low‐tension’ glaucoma [LTG]) and a warmer/redder colour indicates higher IOP (‘high‐tension’ glaucoma [HTG]). Note that the colour steps within HTG and LTG groups are not uniform, but instead are defined by the limits of IOP found in the present study.

In addition to the categorical divisions of very low‐, middling low‐ and high‐tension glaucoma described in Figure 6, we also generated a version of the biplot with each data point representing a specific intraocular pressure measurement (Figure 7). This enabled us to visualise further the potential relationship across a continuum of intraocular pressure (rather than by partitioned, discrete categories which could mask trends). The results in Figure 7 provide further evidence of the heterogeneity in structural and functional measurements among individual patients and emphasise that the separability results from Figures 4 and 6, when comparing partitioned groups, were due to cohort‐level effects.

## DISCUSSION

In this study, we applied principal components analysis to determine whether quantitative clinical parameters could be used to distinguish patients newly diagnosed with high‐ and low‐tension glaucoma using a common intraocular pressure criterion. When using the 21 mm Hg cut‐off criterion, we found no meaningful separability between high‐ and low‐tension glaucoma patients, with substantial overlap in clinical features between the cohorts at the time of diagnosis. Further separating the groups into three intraocular pressure strata showed greater separability between groups, but the overlap remained, and further separating by individual intraocular pressure measurements also returned no appreciable distinction. In both analyses, the largest contributors explaining these differences were mainly optic nerve head and retinal nerve fibre layer structural parameters. These results highlight the inability of quantitative clinical parameters, as expressed using common clinical outputs on optical coherence tomography and standard automated perimetry, to meaningfully distinguish between high‐ and low‐tension glaucoma at the time of the initial glaucoma diagnosis.

### The role of intraocular pressure in clinical phenotyping open‐angle glaucoma

Despite not being part of the formal definition of glaucoma, intraocular pressure remains an integral part of glaucoma management. The EMGT demonstrated that low‐tension glaucoma tends to have slower visual field progression than high‐tension glaucoma when comparing their natural histories.^7^, ^44^ This information would be useful for prognostication and setting treatment expectations and remains relevant for risk titration in the case of ocular hypertension.^45^ Furthermore, reducing intraocular pressure remains the sole evidence‐based therapeutic management strategy for glaucoma, irrespective of the pressure‐defined phenotype.^1^, ^46^, ^47^, ^48^ Additionally, some studies have suggested that patients with lower baseline intraocular pressure are less likely to benefit from selective laser trabeculectomy compared with those with higher baseline pressure levels.^49^ Thus, intraocular pressure remains an important consideration in patient management, and we emphasise that the purpose of this exercise was not to diminish the value of intraocular pressure as part of the assessment but to understand structural and functional differences in clinical practice.

### Differences in functional loss between high‐ and low‐tension glaucoma

The risk of glaucoma delineated by intraocular pressure level has been well‐studied. However, differences in functional loss, either qualitatively (such as the pattern of the defect) or quantitatively (such as severity or depth), remain debated. An earlier study by Motolko et al.^50^ found no significant differences in visual field indices between high‐ and low‐tension glaucoma using kinetic perimetry and was supported by other studies that used different forms of perimetry.^51^ For instance, King et al.^52^ used Octopus perimetry and found similar quantitative characteristics in high‐ and low‐tension glaucoma patients. In combination with the present results, there is evidence suggesting that global measures of functional loss are similar between high‐ and low‐tension patients. However, our results demonstrate that the overall effect size was small, and thus, these differences are insufficient to separate patients meaningfully into high‐ and low‐tension glaucoma groups.

Thonginnetra et al.^51^ found that although low‐tension glaucoma and high‐tension glaucoma patients had similar levels of Humphrey Field Analyzer global index values, more patients with low‐tension glaucoma presented with centralised defects and deeper scotoma compared with high‐tension glaucoma. This has been replicated by several studies^52^, ^53^, ^54^, ^55^ and debated by others.^56^ Park et al.^57^ examined glaucoma patients with initial parafoveal and peripheral scotomas, where patients with lower maximum untreated intraocular pressure tended to have more initial parafoveal visual field loss compared with those with higher intraocular pressures. This approach was binarised with an a priori method of separating parafoveal and peripheral scotomata. Our approach allowed us to examine a potential spectrum of central visual field loss by the number of affected central test locations, and we found no significant difference between newly diagnosed high‐ and low‐tension glaucoma patients. This notably differs from previous studies which have suggested that centrally located defects are more prevalent in low‐tension cases compared with high‐tension glaucoma.^11^, ^58^ We used a consecutive sampling strategy across 5 years of clinical data to minimise the amount of systematic and selection bias within our clinic. Furthermore, the high‐ and low‐tension glaucoma cohorts had no significant difference in mean deviation values, indicating similar functional severity in the context of the disease (see below for further discussion) and the lack of a systematic bias in our findings. However, the present patient cohort represents those seen within our health care setting and may not be generalisable to other contexts.

### Differences in structure loss between high‐ and low‐tension glaucoma

Another suggested difference between high‐ and low‐tension glaucoma relates to the structural findings. Studies using confocal scanning laser topography have had varying results, with some suggesting that low‐tension glaucoma exhibits greater retinal nerve fibre layer loss,^59^, ^60^, ^61^ while others show no significant differences between phenotypes.^62^

Several studies using qualitative spectral‐domain optical coherence tomography measurements have shown more consistent results compared with those conducted with Heidelberg Retinal Tomography (HRT), with low‐tension glaucoma having a tendency for more focal loss within the macula compared with high‐tension glaucoma.^8^, ^14^, ^34^, ^63^ For example, Kim et al.^14^ used a quantitative percentage method to demonstrate differences in focal and global structural losses between high‐ and low‐tension glaucoma. Although statistically significant, there was substantial overlap in the distributions of the retinal nerve fibre layer and ganglion cell complex measurements. Firat et al.^34^ found that high‐tension glaucoma patients exhibited thinner macula and retinal nerve fibre layer thickness values compared with low‐tension glaucoma patients. However, in terms of localised parameters, only the superior retinal nerve fibre layer thickness value discriminated between phenotypes.

However, the challenge in interpreting differences in structure or function between phenotypes is in suitably matching comparative cohorts, as matching by the severity of disease requires fundamental assumptions in the ground truth for determining disease stage. For example, the same level of mean deviation between patients may manifest with regions of visual field loss with different depths (diffuse vs. localised). A different challenge is the assumption that patients are matched based on their stage of disease, but studies using patients with established glaucoma may be capturing results at different points of the disease process. Using results from newly diagnosed patients may be helpful in partly mitigating this limitation. Our approach leverages the structure–function relationship by deriving principal components that highlight correlated quantitative metrics. Our results support the notion that, at the time of diagnosis, there is significant overlap in quantitative values between high‐ and low‐tension glaucoma and that there are inherent correlations between subsets of parameters that might otherwise be spuriously identified as significantly discriminatory.

### Sub‐categorisation of low‐tension glaucoma

Kim et al.^11^ found that low‐tension glaucoma patients with lower intraocular pressure (<15 mm Hg) exhibited lower mean deviation and pattern standard deviation compared with those with relatively higher intraocular pressures. Additionally, they reported that retinal nerve fibre layer defects in the lower intraocular pressure group appeared more localised to the macula.^11^ Wang et al.^9^ observed that patients with lower teen intraocular pressures exhibited less structural damage compared with those with relatively higher intraocular pressure. Both studies highlighted the possibility of a spectrum or ‘gradient’ of intraocular pressure‐related structural or functional changes. Pathophysiologically, higher intraocular pressure would be expected to result in worse structural and functional findings.^64^ Our findings, at face value, support this postulation, with subtle differences between three pressure‐defined groups. Simultaneously, the differences were small, with substantial overlap between groups. We chose to represent these findings using a 2D biplot to visualise the distributions when based on the two main principal components, as per our previous methods.^23^ Since the first two principal components accounted for no more than 60% of the total variance, it is possible that the third principal component could contribute to greater separation. However, even when plotting and calculating the centroid distance in 3D space, there was no appreciable increase in separability, further supporting our initial conclusions when using only principal components 1 and 2. This could be due to the relatively small increments over which intraocular pressure is measured and the phenotypes are defined, such that measurement variability^65^ could affect group allocation. Similarly, the intraocular pressure measured at one visit may not represent the patient's ground truth result, due to intra‐ or inter‐day variability.^18^ Another reason for the overlap may be due to the use of quantitative, global parameters for analysis in the present study. For instance, each mean deviation level could indicate a range of different visual field patterns, including nasal, arcuate, paracentral and generalised defects. In conjunction with other proposals^11^, ^50^, ^52^, ^57^ regarding differences in presenting patterns of defect across pressure‐defined phenotypes, patients with different scotomata may therefore demonstrate overlap in their quantitative metrics.

### Limitations

We used a single clinical centre for collecting data and used a strict criterion for only including newly diagnosed glaucoma patients in the study, and thus the separability results are generalisable only to our cohort. Similarly, we focussed on using quantitative data from clinician‐facing outputs from the Humphrey Field Analyzer and Cirrus optical coherence tomography, which again limits our generalisability to the outputs from these devices only. For instance, we used a 24‐2 visual field grid for our visual field quantitative metrics, which may miss some central defects that might be detected when using the 10‐2 or 24‐2C protocols.^66^, ^67^, ^68^ As described in the literature, there may be qualitative, morphological differences between pressure‐defined phenotypes, which are not conducive to the analysis method used in the present study that might benefit from archetypal analysis examining patterns of loss, both structurally and functionally.^69^, ^70^

Additionally, our data set consisted of cross‐sectional results which were retrospectively collected from an existing database. Although biases such as observational and recall do not necessarily apply in this case (as the data were initially recorded for clinical purposes and were subsequently locked into the electronic medical record), two significant limitations of cross‐sectional data that may impact results are the distinction between causation and association and the reduced sampling of uncommon presentations or covariates (see below).^71^ Similarly, while there is an advantage of reduced observer bias (as data were not specifically collected for the study), a limitation is that some data of interest may be absent, limiting the factors available for analysis.^71^ The addition of longitudinal data could provide additional insights into the relationships between pressure‐defined phenotypes. This would require a different study design and data set. Accordingly, we make no conclusions regarding progression rates, which may also differ between phenotypes.

A further limitation of this study was the possible under‐sampling of potential covariates of glaucoma, such as obstructive sleep apnoea and migraines. When performing our initial factor analysis, we excluded obstructive sleep apnoea to avoid recall bias, though other covariates could have also been similarly affected. Alternative methods for examining covariates related to medical history require a different study design.

Finally, the glaucoma subjects used in the present study were extracted from a clinical glaucoma service. Although we required three clinicians to separately agree on the diagnosis, we did not use more stringent data requirements for defining glaucoma, such as multiple confirmatory visual field tests, overt disease progression or 10‐2 visual field testing. Similarly, we did not require diurnal intraocular pressure measurements to be taken for group assignment. In clinical practice, there is diversity in glaucoma diagnosis and management among clinicians, and there is the possibility of reduced specificity for the purposes of a research study. Specifically, we also did not require the presence of visual field defects (i.e., pre‐perimetric glaucoma patients were included) to reflect current clinical diagnostic paradigms. While we recognise that the reduced specificity of the present cohort may affect statistical power, we believe that the findings remain relevant to current clinical practice, until further guidance is provided. Perimetric indices related to variability, progression and 10‐2 outputs would be interesting to investigate in a future study using a similar framework as proposed here. Similarly, other techniques such as corneal biomechanics and ocular blood flow would be contributory to future models.

## CONCLUSION

Using the present principal components analysis framework, we found that no quantitative clinical parameters could meaningfully discriminate between newly diagnosed high‐tension and low‐tension patients. Although we observed small differences between patients across pressure‐defined phenotypes, there was a large degree of overlap in all quantifiable structural and functional parameters. Our findings, in principle, support the proposal of some potential differences in structural and functional loss across a spectrum of intraocular pressure at the cohort level, but a meaningful distinction of quantitative outputs in clinical practice at the time of diagnosis is unlikely to be achieved due to the heterogeneity of presentation at the individual level.

## AUTHOR CONTRIBUTIONS

**Daniel Rafla:** Formal analysis (equal); investigation (equal); validation (equal); visualization (equal); writing – original draft (equal); writing – review and editing (equal). **Sieu K Khuu:** Formal analysis (equal); methodology (equal); validation (equal); visualization (equal); writing – review and editing (equal). **Sahana Kashyap:** Data curation (supporting); formal analysis (supporting); investigation (supporting). **Michael Kalloniatis:** Conceptualization (equal); formal analysis (equal); funding acquisition (lead); project administration (equal); resources (equal); supervision (equal); validation (equal); visualization (equal); writing – review and editing (equal). **Jack Phu:** Conceptualization (lead); data curation (equal); formal analysis (equal); investigation (equal); methodology (equal); project administration (lead); supervision (equal); validation (equal); visualization (equal); writing – review and editing (equal).

## FUNDING INFORMATION

This work was supported in part by the National Health and Medical Research Council of Australia Ideas Grant to MK, SK and JP (NHMRC 1186915), and a Beta Sigma Kappa Fellowship to JP. In addition, DR was supported, in part, by a scholarship from Guide Dogs NSW/ACT as were the salaries of MK and JP. Clinical services at the Centre for Eye Health are primarily supported by Guide Dogs NSW/ACT. The funding bodies had no role in the conceptualisation or decision to disseminate the present work.

## CONFLICT OF INTEREST STATEMENT

All authors declare no conflicts of interest.

## Supporting information


Figures S1–S4.
