# Supplementary material for: Visualising structural and functional characteristics distinguishing between newly diagnosed high-tension and low-tension glaucoma patients
Source: Ophthalmic Physiol Opt. 2023 Mar 25;43(4):771–87. doi: 10.1111/opo.13129 (PMC10946885; doi:10.1111/opo.13129)
Supplement: Supplementary file 1 — Supplementary files (DOCX 4.30 MB) [file 44402_2023_4304017_MOESM1_ESM.docx]

**Visualising structural and functional characteristics distinguishing between newly-diagnosed high-tension and low-tension glaucoma patients**

**Supplementary material**

Rafla, Daniel^1,2^; Khuu, K., Sieu^2^; Kashyap, Sahana^1,2^; Kalloniatis, Michael^1,2,3^; Phu, Jack^1,2,4,5^

1. Centre for Eye Health, The University of New South Wales, UNSW, Sydney, 2052, Australia.

2. School of Optometry and Vision Science, The University of New South Wales, UNSW, Sydney, 2052, Australia.

3. School of Medicine (Optometry), Deakin University, Waurn Ponds, 3216, Australia.

4. Faculty of Medicine, University of Sydney, Sydney, 2006, Australia.

5. Concord Clinical School, University of Sydney, Sydney, 2006 Australia

Financial disclosures: This work was supported in part by the National Health and Medical

Research Council of Australia Ideas Grant to MK , SK, and JP (NHMRC 1186915), and a Beta Sigma Kappa Fellowship to JP. In addition, DR was supported, in part, by a scholarship from Guide Dogs NSW/ACT as were salaries of MK and JP. Clinical services at the Centre for Eye Health are primarily supported by Guide Dogs NSW/ACT. The funding bodies had no role in the conceptualisation or decision to disseminate the present

work.

Commercial Relationship disclosure: D Rafla, None; S Khuu, None; S Kashyap, None; M Kalloniatis, None; J Phu, None


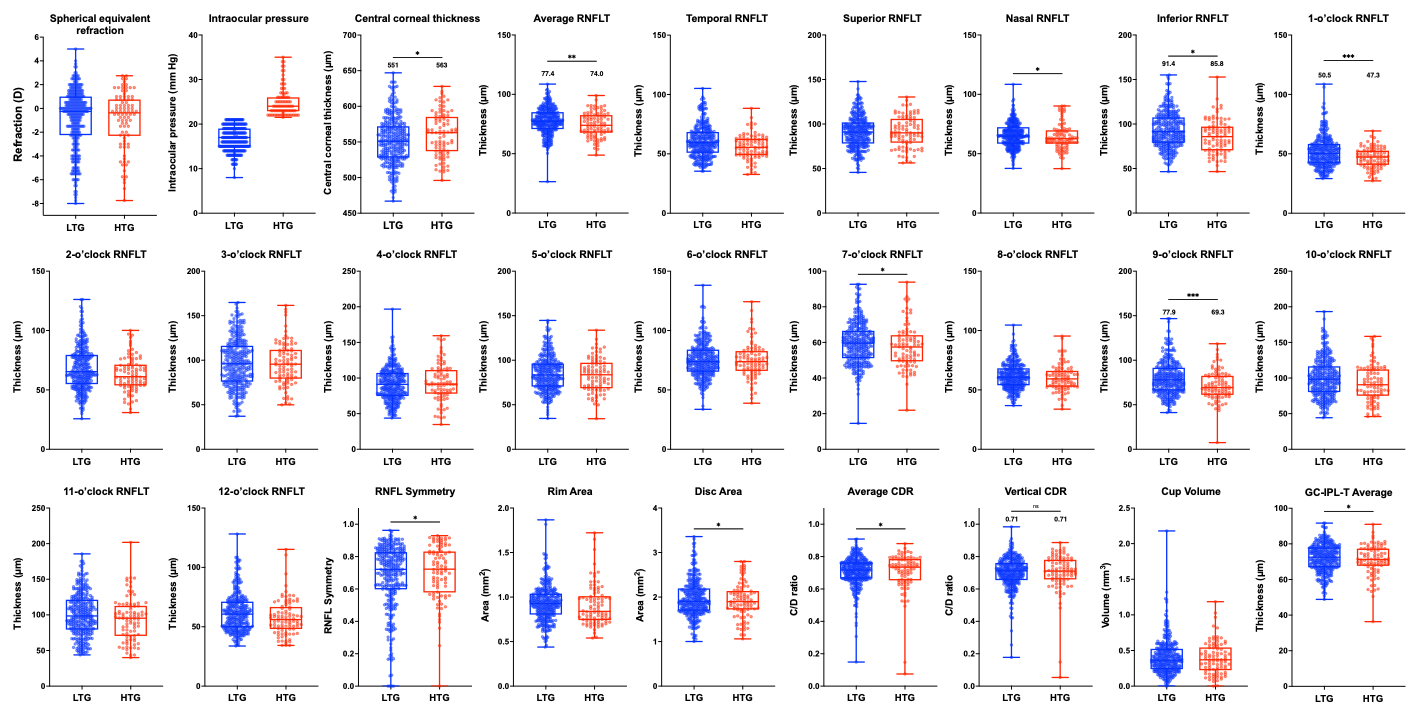

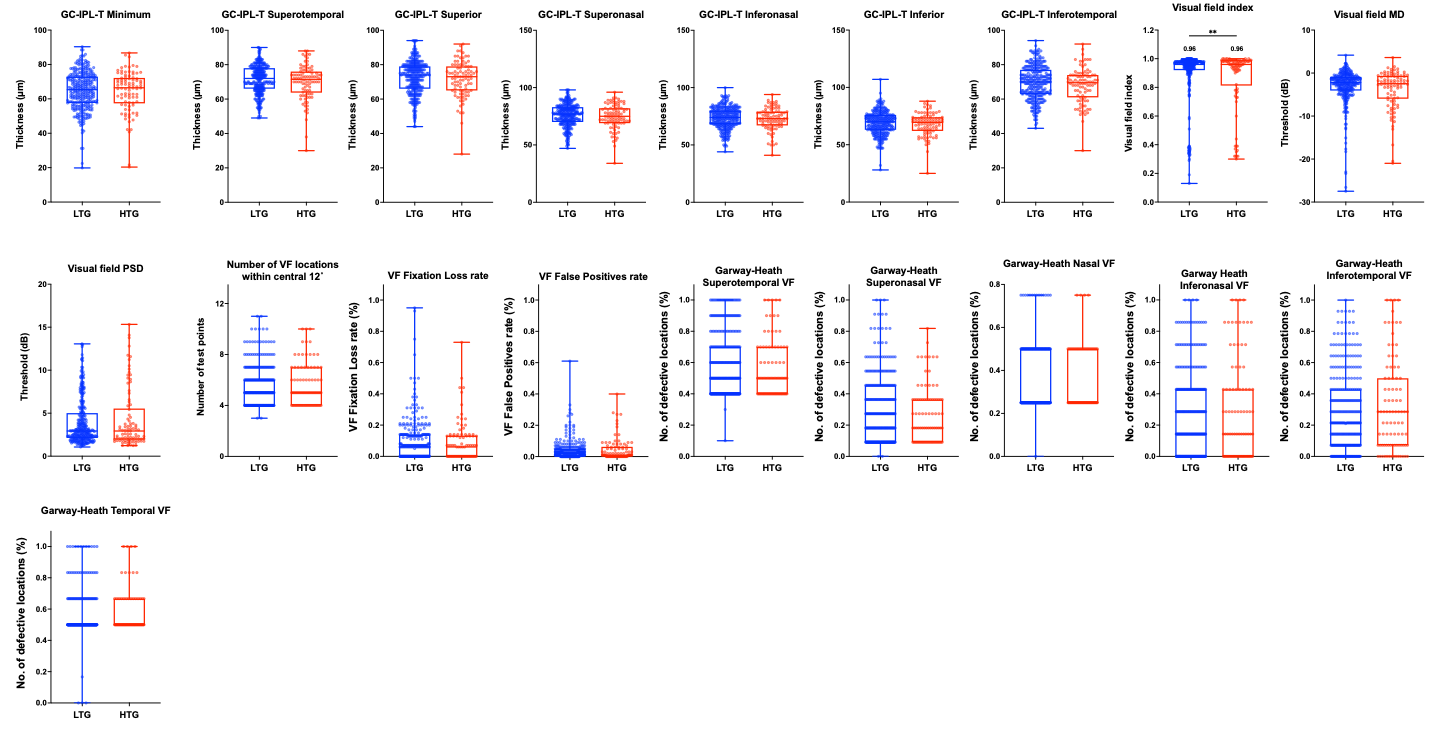
**Supplementary Figure 1:** Box-and-whisker plot showing the median, interquartile and full range results of all extracted quantitative clinical parameters for all low-tension (LTG = blue) and high-tension (HTG = red) glaucoma patients (where * = p < 0.05, ** = p < 0.01, *** = p < 0.001). RNFLT = retinal nerve fibre layer thickness. GC-IPL-T = ganglion cell-inner plexiform layer thickness. CDR = cup-to-disc ratio.

**
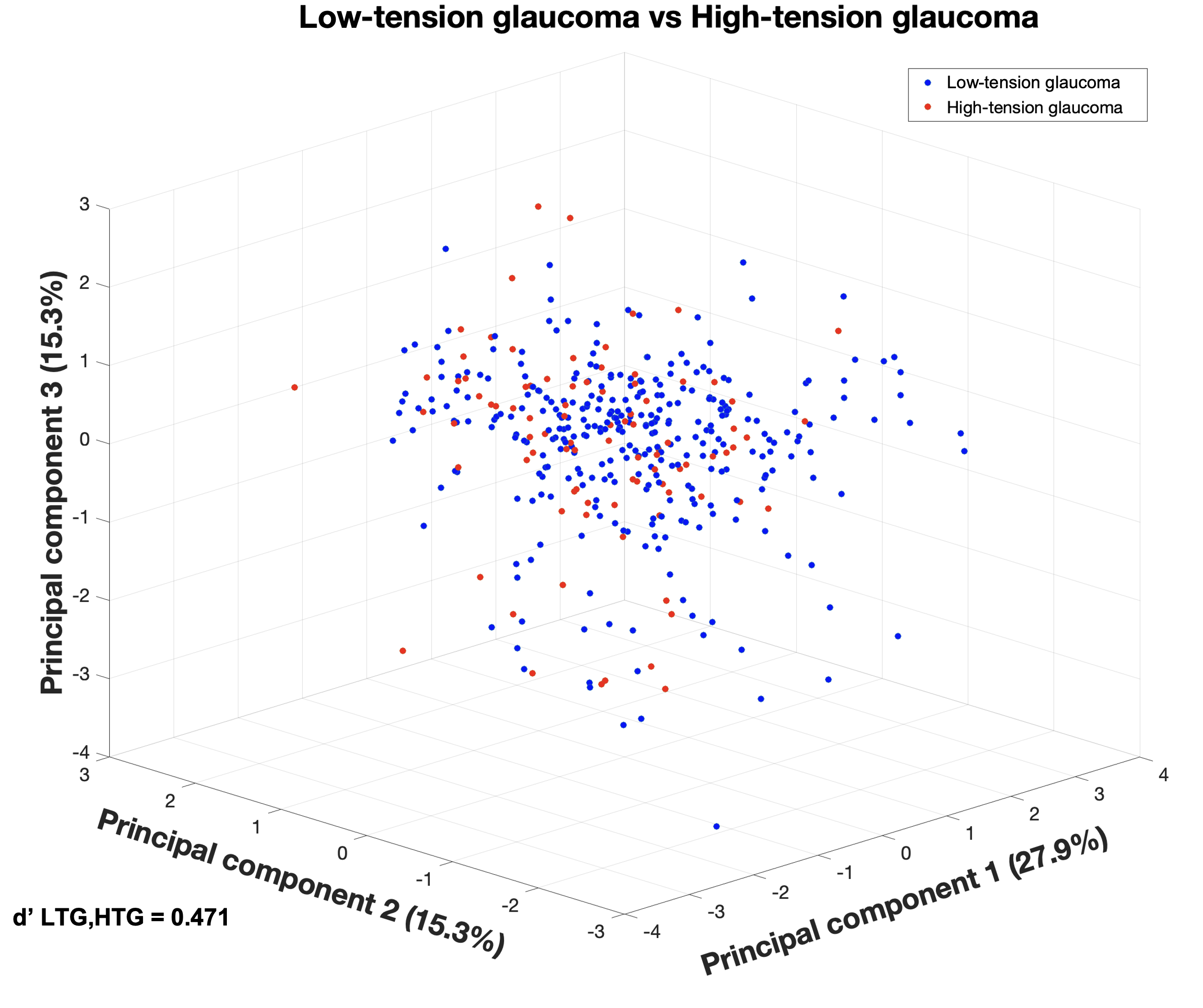
**

**Supplementary Figure 2:** 3D plot mapping individual patient results comparing principal components 1,2 and 3 for all low-tension and high-tension glaucoma subjects, where principal components 1,2 and 3 are shown on the x-, y- and z-axes respectively. The blue circles represent patients with normal-tension (NTG) and red circles indicate high tension glaucoma (HTG). The separability between centroids (d(x,y,z)) is shown in the inset.


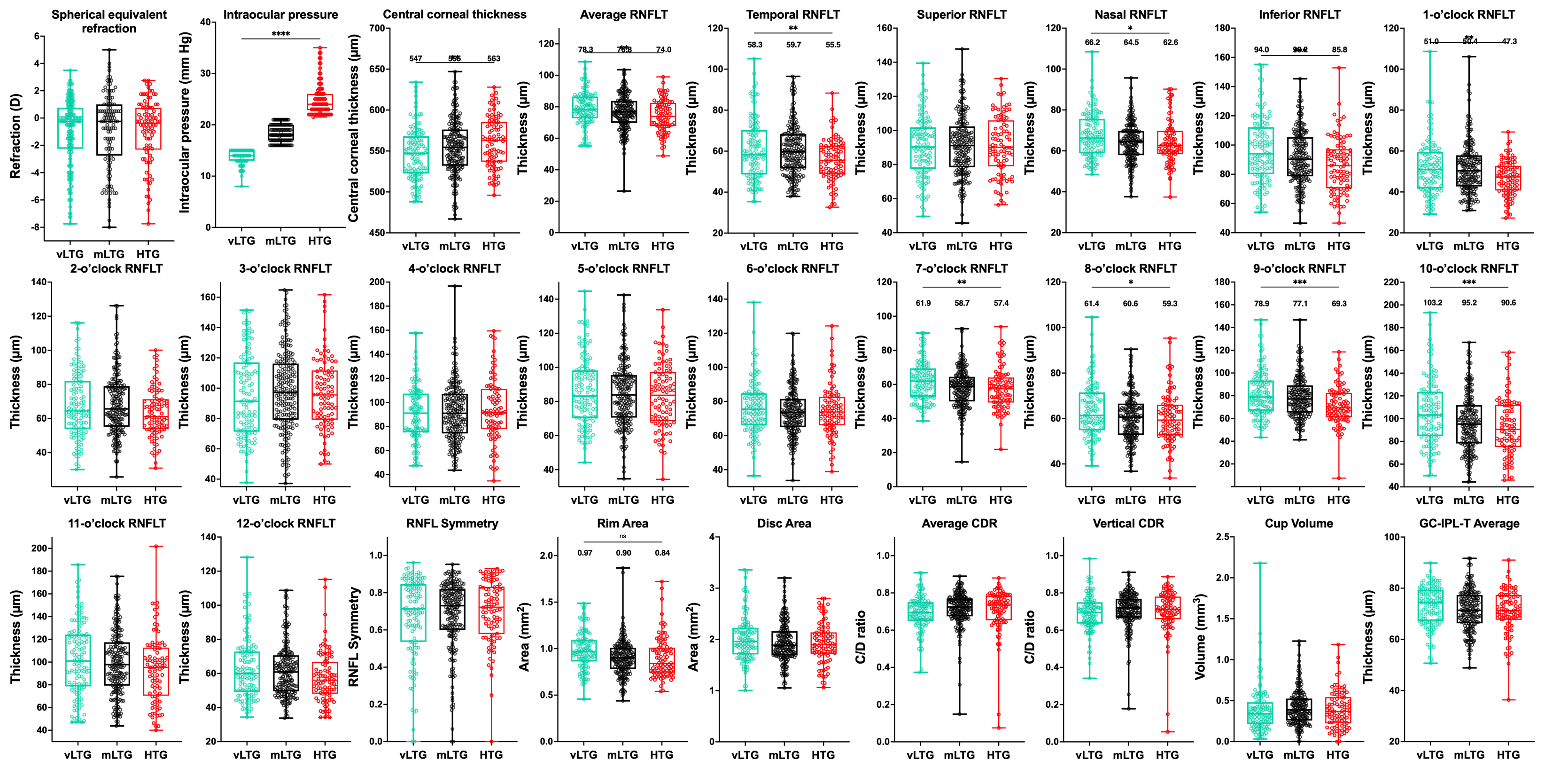

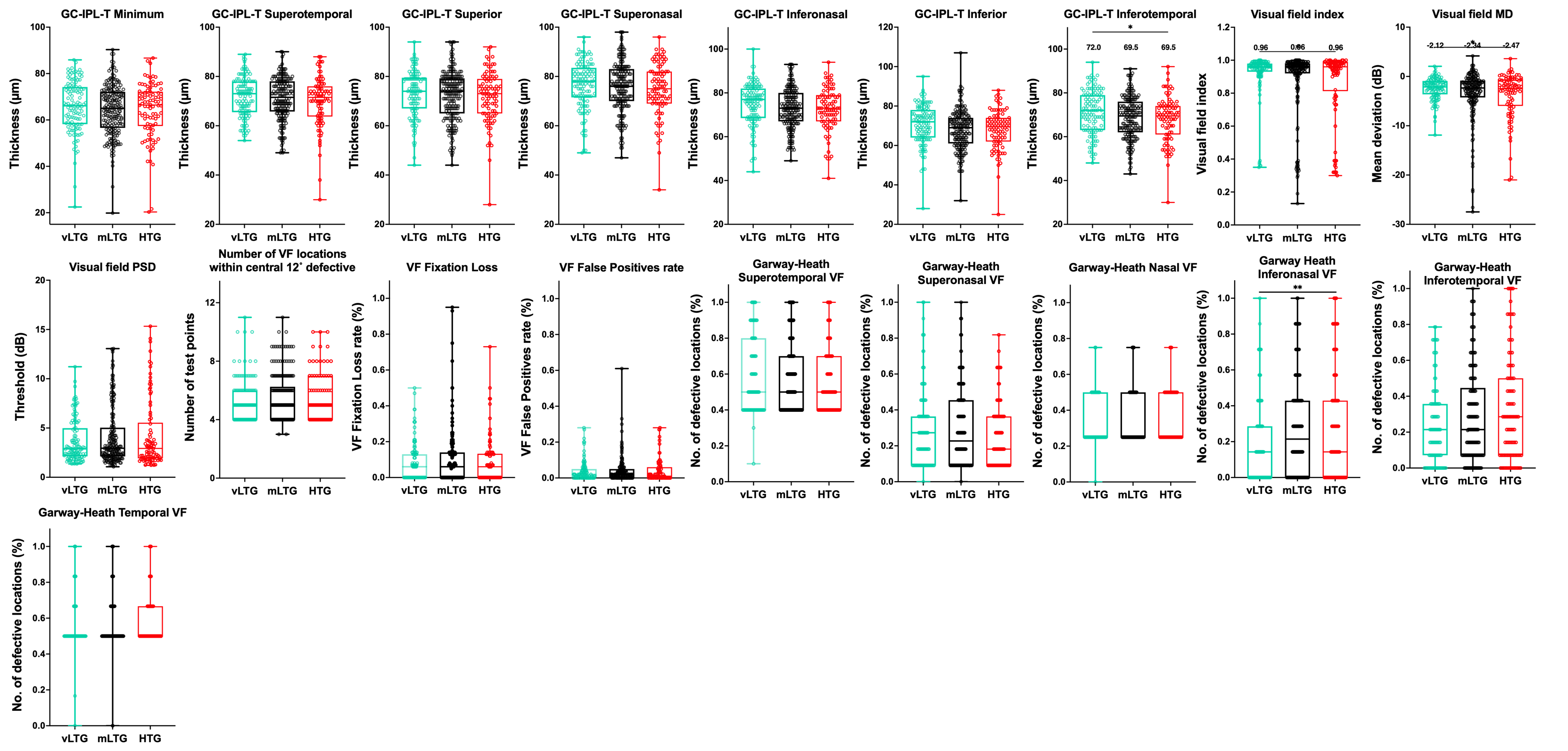


**Supplementary Figure 3:** Box-and-whisker plot showing the median, interquartile and full range of individual results in all extracted quantitative clinical parameters for very low-tension (LTG = aqua), middling low-tension(mLTG = black) and high-tension (HTG = red) glaucoma patients (where * = p < 0.05, ** = p < 0.01, *** = p < 0.001 for the multiple comparisons analysis of the ANOVA). RNFLT = retinal nerve fibre layer thickness. GC-IPL-T = ganglion cell-inner plexiform layer thickness. CDR = cup-to-disc ratio.


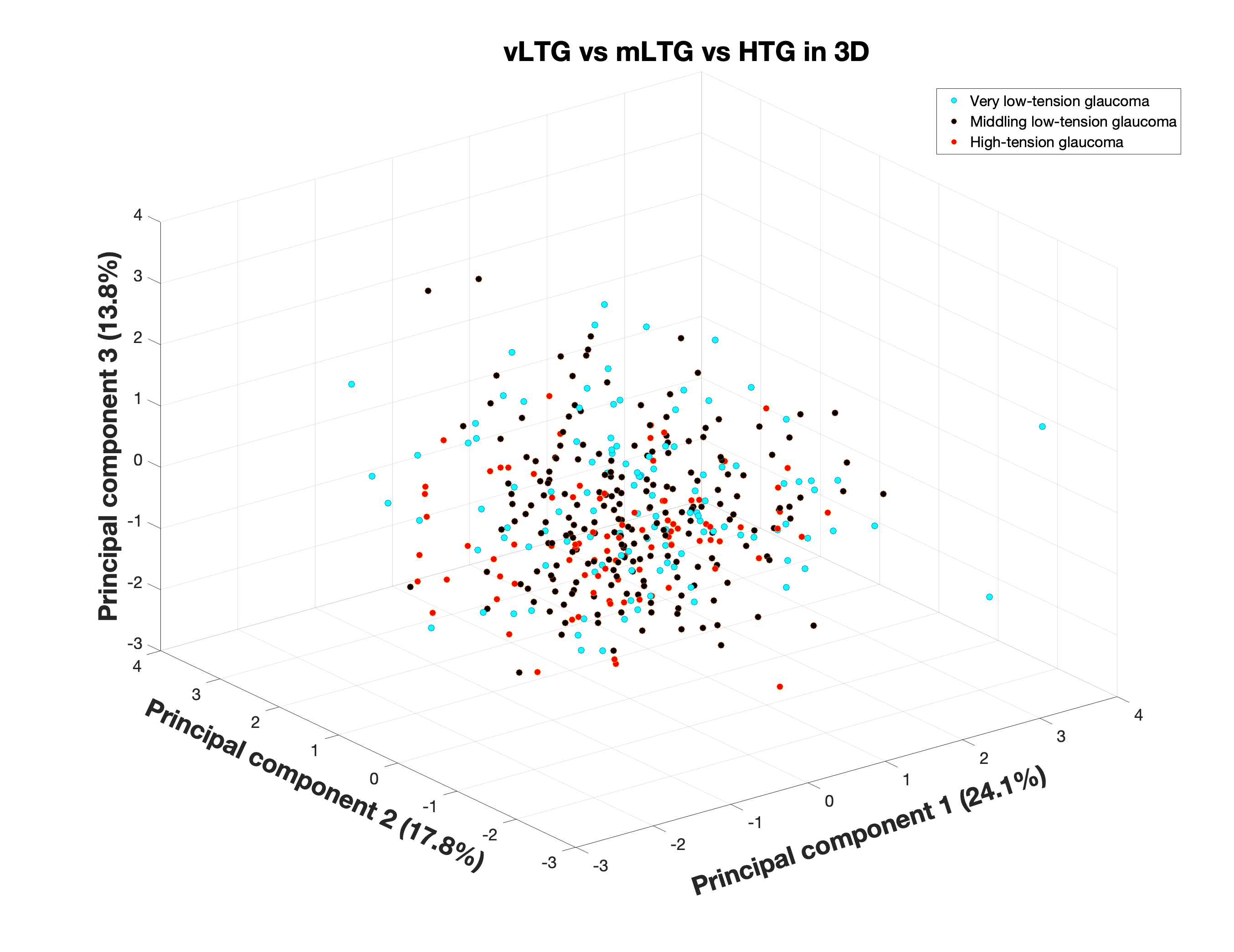


**d’ vLTG, mLTG = 0.652**

**d’ vLTG, HTG = 0.438**

**d’ mLTG, HTG = 0.405**

**Supplementary Figure 4:** 3D plot mapping individual patient results comparing principal components 1,2 and 3 for all normal-tension and high-tension glaucoma subjects, where principal components 1,2 and 3 are shown on the x-, y- and z-axes respectively. The light-blue circles represent patients with very low-tension (vLTG), the black circles represent patients with middling low-tension glaucoma(mLTG) and red circles indicate patients with high tension glaucoma (HTG). The separability between centroids (d(x,y,z)) is shown in the inset.
